# Screening and management of mental health and substance use disorders in HIV treatment settings in low‐ and middle‐income countries within the global IeDEA consortium

**DOI:** 10.1002/jia2.25101

**Published:** 2018-03-30

**Authors:** Angela M Parcesepe, Catrina Mugglin, Fred Nalugoda, Charlotte Bernard, Evy Yunihastuti, Keri Althoff, Antoine Jaquet, Andreas D Haas, Stephany N Duda, C William Wester, Denis Nash, the International epidemiology to Evaluate AIDS (IeDEA) Consortium

**Affiliations:** ^1^ University of North Carolina at Chapel Hill Gillings School of Global Public Health Chapel Hill NC USA; ^2^ Institute for Implementation Science in Population Health City University of New York New York NY USA; ^3^ Institute of Social and Preventive Medicine University of Bern Bern Switzerland; ^4^ Rakai Health Sciences Program Kalisizo Uganda; ^5^ Bordeaux Population Health Research Center University of Bordeaux INSERM Bordeaux France; ^6^ INSERM ISPED Bordeaux Population Health Research Center Bordeaux France; ^7^ Faculty of Medicine Universitas Indonesia/Cipto Mangunkusumo Hospital Jakarta Indonesia; ^8^ Johns Hopkins Bloomberg School of Public Health Baltimore MD USA; ^9^ Department of Biomedical Informatics Vanderbilt University Medical Center Nashville TN USA; ^10^ Department of Medicine Division of Infectious Diseases Vanderbilt University Medical Center Nashville TN USA; ^11^ Vanderbilt Institute for Global Health (VIGH) Nashville TN USA

**Keywords:** mental health, HIV, integration, treatment, depression, PTSD

## Abstract

**Introduction:**

Integration of services to screen and manage mental health and substance use disorders (MSDs) into HIV care settings has been identified as a promising strategy to improve mental health and HIV treatment outcomes among people living with HIV/AIDS (PLWHA) in low‐ and middle‐income countries (LMICs). Data on the extent to which HIV treatment sites in LMICs screen and manage MSDs are limited. The objective of this study was to assess practices for screening and treatment of MSDs at HIV clinics in LMICs participating in the International epidemiology Databases to Evaluate AIDS (IeDEA) consortium.

**Methods:**

We surveyed a stratified random sample of 95 HIV clinics in 29 LMICs in the Caribbean, Central and South America, Asia‐Pacific and sub‐Saharan Africa. The survey captured information onsite characteristics and screening and treatment practices for depression, post‐traumatic stress disorder (PTSD), substance use disorders (SUDs) and other mental health disorders.

**Results:**

Most sites (n = 76, 80%) were in urban areas. Mental health screening varied by disorder: 57% of sites surveyed screened for depression, 19% for PTSD, 55% for SUDs and 29% for other mental health disorders. Depression, PTSD, SUDs and other mental health disorders were reported as managed on site (having services provided at the HIV clinic or same health facility) at 70%, 51%, 41% and 47% of sites respectively. Combined availability of screening and on‐site management of depression, PTSD, and SUDs, and other mental health disorders was reported by 42%, 14%, 26% and 19% of sites, respectively. On‐site management of depression and PTSD was reported significantly less often in rural as compared to urban settings (depression: 33% and 78% respectively; PTSD: 24% and 58% respectively). Screening for depression and SUDs was least commonly reported by HIV programmes that treated only children as compared to HIV programmes that treated only adults or treated both adults and children.

**Conclusions:**

Significant gaps exist in the management of MSDs in HIV care settings in LMICs, particularly in rural settings. Identification and evaluation of optimal implementation strategies to scale and sustain integrated MSDs and HIV care is needed.

## 1 Introduction

Mental health and substance use disorders (MSDs) are highly prevalent among persons living with HIV/AIDS (PLWHA) globally, including in low‐ and middle‐income countries (LMICs) and more prevalent among PLWHA compared to the general population 1, 2. According to the Diagnostic and Statistical Manual of Mental Disorders, Fifth Edition (DSM‐5), mental disorders are “characterized by clinically significant disturbance in an individual's cognition, emotion regulation, or behaviour that reflects a dysfunction in the psychological, biological, or developmental processes underlying mental functioning”^3^. Common mental disorders include depression and post‐traumatic stress disorder (PTSD). As defined by the DSM‐5, substance use disorders (SUDs) involve a “cluster of cognitive, behavioural and psychological symptoms indicating that the individual continues using the substance despite significant substance‐related problems” 3. SUDs include alcohol and drug use disorders. Approximately 50% of PLWHA in LMICs meet diagnostic criteria for one or more MSDs 1, 2.

Poor mental health has been associated with suboptimal HIV treatment outcomes throughout the HIV care cascade, including late initiation of combination antiretroviral therapy (ART), poor ART adherence and lack of viral suppression 4, 5, 6, 7. MSDs remain under‐diagnosed and under‐treated in many LMICs. Because MSDs are common and drive suboptimal outcomes throughout the HIV care cascade, untreated MSDs may serve as significant, but modifiable barriers to optimal HIV treatment.

Evidence‐based management for many MSDs significantly improves mental health outcomes 8, 9, 10, 11. Integration of evidence‐based interventions to manage MSDs into HIV care has been identified as a promising strategy to improve mental health and HIV treatment outcomes of PLWHA in LMICs 12, 13. The World Health Organization (WHO) recommends integration of or linkage to mental health services for PLWHA, where possible, and has developed the Mental Health Gap Action Programme (mhGAP) to provide evidence‐based guidelines for the diagnosis and management of priority mental health disorders in routine healthcare settings 14, 15. The extent to which screening and management of MSDs is integrated into HIV treatment programmes in LMICs remains largely unknown 1, 16. The objective of this study was to describe MSD screening and management practices in a representative sample of HIV treatment sites in LMICs participating in the International epidemiology Databases to Evaluate AIDS (IeDEA) consortium.

## 2 Methods

**Figure 1:**
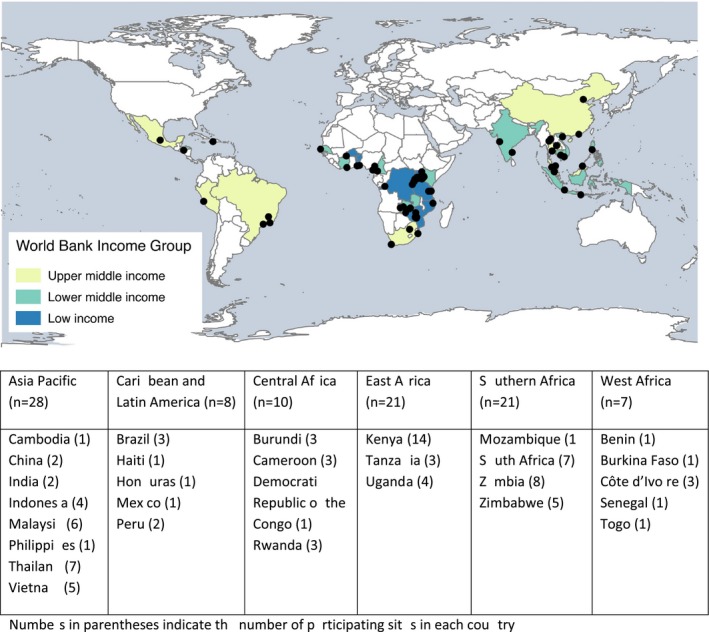
Geographic distribution of the 95 HIV treatment sites from the IeDEA network participating in the MSD survey.

The IeDEA consortium (iedea.org) is a global research consortium including HIV care and treatment programmes from North America, the Caribbean, Central and South America, Asia‐Pacific and sub‐Saharan Africa 17, 18, 19. IeDEA is funded by the U.S. National Institutes of Health to provide a resource for globally diverse clinical HIV/AIDS observational data 18, 19. Our survey was conducted with a stratified random sample of 95 HIV treatment sites in 29 LMICs in the Caribbean, Latin America, the Asia‐Pacific region and sub‐Saharan Africa (See Figure 1). To participate in IeDEA, HIV care and treatment sites must be located in one of the countries in which IeDEA is currently operating and have the capacity to routinely contribute electronic data to the IeDEA consortium. In this way, IeDEA sites may have more resources and be more highly functioning than sites in that country that are not participating in IeDEA. Based on previous IeDEA sites assessments, IeDEA sites represent various levels of the health system, are privately and publicly funded, and are located in urban and rural areas 20, 21.

### 2.1 Survey development

IeDEA investigators, in collaboration with the President's Emergency Plan for AIDS Relief Non‐Communicable Diseases (PEPFAR NCD) working group, developed a survey to assess integration of screening and treatment practices for numerous non‐communicable diseases (NCDs) into HIV care, including MSDs. The current analysis is limited to assessment of the integration of screening and management practices for MSDs.

### 2.2 Sampling frame

This study surveyed a stratified random sample of HIV treatment programmes across the six low‐ and middle‐income IeDEA regions: the Caribbean, Central and South America, Asia‐Pacific, Central Africa, East Africa, Southern Africa and West Africa. When possible, the sampling frame was stratified according to the following site characteristics: setting (i.e. urban, rural), patient population (i.e. adults only, children/adolescents only, adults and children/adolescents), and level of care (i.e. health centre; district hospital; regional, provincial, or university hospital) at the country level. However, stratification by the three stratification variables was not possible for all countries (e.g. all IeDEA sites in Côte d'Ivoire are urban). Stratification variables were obtained from the most recent site assessment 21 and verified during implementation of the current survey. A random sampling strategy was used so that sites selected would be representative of clinics located in LMICs within IeDEA. Eligible sites were: (1) HIV treatment programmes; (2) current members of the IeDEA consortium; and (3) located in one of the six low‐ and middle‐income IeDEA regions.

### 2.3 Data collection and statistical analyses

Data collection occurred between August 2016 and May 2017. English and French versions of the survey were available online and as paper‐based instruments. The online version was implemented using REDCap (projectredcap.org), a secure web‐based application hosted by the University of Bern 22. IeDEA research coordinators distributed surveys to sites in their region. Survey instructions explained that the survey should be completed by individual(s) knowledgeable about clinic capacity and services offered and encouraged the respondent to contact other people at the site if they did not know or were uncertain about answers to survey questions. Surveys completed on paper were entered into REDCap by regional representatives or study staff members. The survey captured information onsite characteristics (level of care, setting, patient population, sector) and screening and management practices for depression, PTSD, SUDs, including alcohol and drug use disorders, and other serious mental illnesses, including schizophrenia and bipolar disorder. Respondents were asked separately about screening and management procedures for: depression, PTSD, SUDs and other serious mental illnesses. The survey was translated into French and sections were back‐translated into English. For each MSD assessed, respondents were asked if their HIV treatment site: screened patients, had a written protocol for screening, how they screened, whom they screened, had a written protocol for management, where management was provided, and the type of staff members responsible for management. Sites and coordinating centres for all IeDEA regions had Institutional Review Board approvals that permitted collection of site‐level data for this survey. This survey collected only site‐level data.

Descriptive statistics summarize the prevalence of screening and management of MSDs overall and by World Bank Income designation of the country in which the site was located. Analyses between site‐level characteristics and screening and treatment of MSDs were conducted using chi‐squared or Fisher's exact tests. Results were presented overall and by World Bank Income Designation (as of July 2015) of the country in which each site was located.

## 3 Results

Of the initial 162 sites approached for participation, 86 sites completed the survey. The main reason for non‐participation was that sites were no longer participating in IeDEA (n = 43; 56%). This attrition occurred over two to three years and represents a challenge of conducting longitudinal research in real‐world service settings. Other reasons for non‐participation included: sites had temporarily suspended operations or were undergoing structural changes (n = 14; 18%), sites had closed down (n = 6; 8%), non‐response (n = 2; 3%), or other reasons (n = 11; 15%). Nine replacement sites were selected and participated resulting in a final sample of 95 sites. Replacement sites were randomly selected from those remaining in that stratum. If no other sites remained in that stratum, no replacement site was chosen. Approximately 43% of respondents described themselves as principal investigators, 26% as head clinician, clinical manager, or nurse, 9% as site manager and 23% as performing another role at the site.

### 3.1 Site characteristics

Among the participating sites, 59 were located in sub‐Saharan Africa, 28 in the Asia‐Pacific region and 8 in the Caribbean and Latin America. The geographical distribution of the 95 participating sites is provided in Figure 1. The majority of sites treated adults and children/adolescents (64%) and were located in urban settings (80%).

#### 3.1.1 Availability of staff members with mental health training

Twenty‐three percent of participating sites reported having staff members trained in psychiatry or psychology at the HIV treatment site. Forty‐two percent reported having such staff members available at their health facility, but not at the HIV treatment site. Among sites that reported having staff members trained in psychiatry or psychology at the HIV treatment site (n = 22), 50% reported that such staff members were physicians, 36% reported that they were mid‐level providers, 32% reported that they were nurses or nursing assistants, 27% reported that they were other types of clinical staff members, and 5% reported that they were non‐clinical staff members.

### 3.2 Screening of MSDs

Fifty‐seven percent of participating sites reported screening HIV patients for depression (Table 1). Twenty‐one percent of sites reported having written guidelines for depression screening. More than one‐third (38%) reported targeted screening of only symptomatic patients while 17% reported screening all patients for depression. Twenty‐eight percent of sites reported screening patients for depression at every visit, 17% at enrolment into care, 6% at ART initiation and 2% reported annual depression screening. Screening for depression was most commonly reported in low‐income countries (72%).

**Table 1:** Screening of mental and substance use disorders in 95 HIV treatment programmes within the IeDEA consortium, overall and by World Bank Income designation

	All n = 95 n (%)	Low‐income countries n = 25 n (%)	Lower middle‐income countries n = 42 n (%)	Upper middle income‐countries n = 28 n (%)
Depression				
Depression screening	54 (57)	18 (72)	24 (57)	12 (43)
Written guidelines in place	20 (21)	9 (36)	7 (17)	4 (14)
Patient population screened				
All patients	16 (17)	5 (20)	7 (17)	4 (14)
Symptomatic patients	36 (38)	13 (52)	15 (36)	8 (29)
Other	1 (1)	0	1 (2)	0
Timing of screening^a^				
Enrolment into care	16 (17)	7 (28)	5 (12)	4 (14)
ART initiation	6 (6)	4 (16)	2 (5)	0
Annually	2 (2)	0	1 (2)	1 (4)
Every visit	27 (28)	14 (56)	10 (24)	3 (11)
Other	20 (21)	3 (12)	11 (26)	6 (21)
Post‐traumatic stress disorder (PTSD)				
PTSD screening	18 (19)	10 (40)	5 (12)	3 (11)
Written guidelines in place	10 (11)	7 (28)	2 (5)	1 (4)
Patient population screened				
All patients	3 (3)	3 (12)	0	0
Patients with symptoms	14 (15)	7 (28)	4 (9)	3 (11)
Other	0	0	0	0
Timing of screening^a^				
Enrolment into care	6 (6)	4 (16)	2 (5)	0
ART initiation	0	0	0	0
Annually	0	0	0	0
Every visit	13 (14)	8 (32)	3 (7)	2 (7)
Other	4 (4)	2 (8)	1 (2)	1 (4)
Substance use disorders				
Substance use disorders screening	52 (55)	12 (48)	27 (64)	13 (46)
Written guidelines in place	19 (20)	6 (24)	7 (17)	6 (21)
Patient population screened				
All patients	29 (31)	5 (20)	17 (40)	7 (25)
Patients with symptoms	19 (20)	7 (28)	8 (19)	4 (14)
Other	4 (4)	0	2 (5)	2 (7)
Timing of screening^a^				
Enrolment into care	28 (29)	4 (16)	16 (38)	8 (29)
ART initiation	6 (6)	2 (8)	3 (7)	1 (4)
Annually	1 (1)	0	0	1 (4)
Every visit	27 (28)	11 (44)	11 (26)	5 (18)
Other	8 (8)	1 (4)	5 (12)	2 (7)
Other mental health disorders				
Other mental health disorders screening	28 (29)	12 (48)	10 (24)	6 (21)
Written guidelines in place	11 (12)	6 (24)	3 (7)	2 (7)
Patient population screened				
All patients	6 (6)	3 (12)	2 (5)	1 (4)
Patients with symptoms	21 (22)	9 (36)	7 (17)	5 (18)
Other	1 (1)	0	1 (2)	0
Timing of screening^a^				
Enrolment into care	7 (7)	3 (12)	2 (5)	2 (7)
ART initiation	4 (4)	2 (8)	2 (5)	0
Annually	0	0	0	0
Every visit	16 (17)	10 (40)	4 (9)	2 (7)
Other	9 (9)	2 (8)	5 (12)	2 (7)

a Categories are not mutually exclusive.

Screening for PTSD was reported by 19% of participating sites. Eleven percent of sites reported having guidelines for PTSD screening. Fifteen percent of sites reported targeted PTSD screening of only symptomatic patients and 3% reported screening all patients for PTSD. Fourteen percent of sites reported screening patients for PTSD at every visit and 6% reported screening patients for PTSD at enrolment into care. Similar to depression, PTSD screening was most commonly reported in low‐income countries (40%).

Half (55%) of sites reported screening for SUDs, including alcohol and drug use disorders. Twenty percent of sites reported having guidelines for SUDs screening. Thirty‐one percent of sites reported screening all patients for SUDs and 20% reported targeted screening of only symptomatic patients. Approximately one‐quarter (28%) of sites reported screening for SUDs at every visit while 29% reported screening for SUDs at enrolment into care. SUDs screening was most commonly reported in lower middle‐income countries (64%).

Twenty‐nine percent of participating sites reported screening patients for other mental health disorders, such as schizophrenia or bipolar disorder. Twelve percent reported that they had written guidelines for screening for other mental health disorders. Twenty‐two percent of clinics reported targeted screening of symptomatic patients for other mental health disorders while 6% reported screening all patients for other mental health disorders. Seventeen percent of clinics reported that they screened for other mental health disorders at every visit.

### 3.3 Management of MSDs

Depression was reported to be managed on site (defined as providing services at the HIV clinic or the same health facility) in 70% of sites (See Table 2). Services to manage depression were reported to be available only off site in 18% of sites and were reported to be unavailable (either on site or off site) in 12% of sites. Individual counselling or group therapy to manage depression was reported to be available on site at 70% and 49% of sites respectively. On‐site management of depression was most commonly reported in upper middle‐income countries (89%) and least commonly reported in lower middle‐income countries (51%).

**Table 2:** Management of mental and substance use disorders at 95 HIV treatment programmes within the IeDEA consortium, overall and by World Bank Income designation

	All n = 95 n (%)	Low‐income countries n = 25 n (%)	Lower middle income‐countries n = 42 n (%)	Upper middle income‐countries n = 28 n (%)
Depression				
Management of depression^a^				
Available on site	64 (70)	18 (78)	21 (51)	25 (89)
Available only off site	17 (18)	3 (13)	12 (29)	2 (7)
Not available	11 (12)	2 (9)	8 (20)	1 (4)
Missing	3	2	1	0
Individual counselling or psychotherapy^a^				
Available on site	66 (70)	20 (83)	21 (50)	25 (89)
Available only off site	15 (16)	1 (4)	11 (26)	3 (11)
Not available	13 (14)	3 (13)	10 (24)	0
Missing	1	1	0	0
Support group or group therapy^a^				
Available on site	46 (49)	16 (67)	12 (28)	18 (64)
Available only off site	19 (20)	1 (4)	13 (31)	5 (18)
Not available	29 (31)	7 (29)	17 (40)	5 (18)
Missing	1	1	0	0
PTSD				
Management of PTSD^a^				
Available on site	45 (51)	14 (61)	9 (23)	22 (85)
Available only off site	29 (33)	6 (26)	19 (49)	4 (15)
Not available	14 (16)	3 (13)	11 (28)	0
Missing	7	2	3	2
Individual counselling or psychotherapy^a^				
Available on site	51 (54)	16 (67)	15 (36)	20 (71)
Available only off site	20 (21)	3 (13)	12 (29)	5 (18)
Not available	23 (24)	5 (21)	15 (36)	3 (11)
Missing	1	1	0	0
Support group or group therapy^a^				
Available on site	40 (43)	14 (58)	12 (29)	14 (50)
Available only off site	20 (21)	3 (13)	11 (27)	6 (21)
Not available	33 (35)	7 (29)	18 (44)	8 (29)
Missing	2	1	1	0
Substance use disorders				
Management of substance use disorders^a^				
Available on site	38 (41)	9 (39)	12 (29)	17 (61)
Available only off site	42 (46)	10 (43)	21 (51)	11 (39)
Not available	12 (13)	4 (17)	8 (20)	0
Missing	3	2	1	0
Other mental health disorders				
Management of other mental health disorders^a^				
Available on site	43 (47)	12 (50)	10 (24)	21 (81)
Available only off site	33 (36)	8 (33)	20 (49)	5 (19)
Not available	15 (16)	4 (17)	11 (27)	0
Missing	4	1	1	2
Written guidelines in place	14 (15)	7 (28)	3 (7)	4 (14)
Availability of psychiatric medications				
Selective serotonin reuptake inhibitors (SSRIs)^a^				
Available on site	49 (52)	8 (32)	16 (39)	25 (89)
Available only off site	24 (26)	10 (40)	14 (34)	0
Not available	21 (22)	7 (28)	11 (27)	3 (11)
Missing	1	0	1	0
Benzodiazepines				
Available on site	77 (81)	19 (76)	32 (76)	26 (93)
Available only off site	7 (7)	4 (16)	3 (7)	0
Not available	11 (12)	2 (8)	7 (17)	2 (7)
Other psychiatric medications^a^				
Available on site	67 (71)	18 (72)	25 (60)	24 (89)
Available only off site	16 (17)	6 (24)	9 (21)	1 (4)
Not available	11 (12)	1 (4)	8 (19)	2 (7)
Missing	1	0	0	1

a Percentages are computed using the number of sites with a non‐missing value.

PTSD was reported to be managed on site in 51% of participating sites. One‐third of sites reported that services to manage PTSD were only available off site, and 16% reported that such services were not available. Individual counselling or psychotherapy to manage PTSD was reported to be available on site at 54% of participating clinics and support groups or group therapy to manage PTSD was reported to be available on site at 43% of participating sites. On‐site management of PTSD was most commonly reported in upper middle‐income countries (85%) and least commonly reported in lower middle‐income countries (23%).

Services to manage SUDs were reported to be available on site at 41% of clinics, only off site at 46% of sites, and were reported to be unavailable at 13% of sites. Services to manage SUDs were more commonly reported in upper middle‐income countries (61%) than in low‐income countries (39%) or lower middle‐income countries (29%).

Services to manage other mental health disorders (e.g. schizophrenia, bipolar disorder) were available on site at 47% of participating sites, were reported to be available only off site in 36% of sites, and were reported to be unavailable at 16% of sites. Selective serotonin reuptake inhibitors (SSRIs), benzodiazepines and other psychiatric medications were reportedly available on site at 52%, 81% and 71% of sites respectively.

On‐site management of all MSDs assessed was significantly more commonly reported among sites that reported having staff members with mental health training. Ninety percent of sites that reported having staff members on site with mental health training reported on‐site management of depression compared to 33% of sites that reported not having staff members on site with mental health training (data not shown). Similarly, 73% of sites that reported having staff members on site with mental health training reported on‐site management of PTSD compared to 16% of sites that reported not having staff members on site with mental health training.

### 3.4 Combined availability of screening and on‐site management of MSDs

Almost half of sites (42%) reported screening and on‐site management of depression (Table 3). In contrast, 14% reported only screening for depression, while 27% of sites reported only on‐site management for depression. Fourteen percent of sites reported both screening and on‐site management of PTSD. Seven percent reported screening, but not on‐site management for PTSD, while 38% of sites reported on‐site management, but not screening for PTSD. One‐quarter (26%) reported screening and on‐site management of SUDs. Thirty percent of sites reported screening, but not on‐site management of SUDs, while 15% of sites reported on‐site management, but not screening for SUDs. Nineteen percent of sites reported both screening and on‐site management of other mental health disorders while 41% reported neither screening nor on‐site management of other mental health disorders.

**Table 3:** Combined availability of screening and on‐site management of mental health and substance use disorders at HIV care and treatment sites within the IeDEA consortium, by mental health or substance use disorder in 2017

	Depression n = 92 n (%)	PTSD n = 88 n (%)	Substance use disorders n = 92 n (%)	Other mental health disorders n = 91 n (%)
Screening and on‐site management available	39 (42)	12 (14)	24 (26)	17 (19)
Only screening available	13 (14)	6 (7)	28 (30)	11 (12)
Only on‐site management available	25 (27)	33 (38)	14 (15)	26 (29)
Neither screening nor on‐site management available	15 (16)	37 (42)	26 (28)	37 (41)

### 3.5 Site characteristics and screening and management of MSDs

Screening of PTSD was significantly less commonly reported in urban compared to rural settings (Table 4). Screening for depression and SUDs was less commonly reported by paediatric only sites than adult sites or sites that treat adults and children/adolescents. On‐site management of depression, PTSD, SUDs and other mental health disorders was associated with level of care of the health facility and patient population treated. On‐site management of depression, PTSD, and other mental health disorders was most commonly reported by regional, provincial or universities hospitals and least commonly reported by health centres. On‐site management of SUDs was most commonly reported by district hospitals and least commonly reported by health centres. On‐site management of all MSDs assessed was most commonly reported by HIV sites that treated adults only and least commonly reported by HIV sites that treated both adults and children/adolescents. On‐site management of depression and PTSD were reported significantly more often in urban settings compared to rural settings. On‐site management of PTSD, SUDs and other mental health disorders was reported significantly more often at public/government‐supported facilities as compared to private facilities.

**Table 4:** Site‐level characteristics and availability of screening and on‐site management of mental health and substance use disorders at 95 HIV treatment sites within the IeDEA consortium, by mental health or substance use disorder in 2017^a^

Characteristic	Total	Screening				On‐site management			
		Depression n (%)	PTSD n (%)	Substance use disorders n (%)	Other mental health disorders n (%)	Depression n (%)	PTSD n (%)	Substance use disorders n (%)	Other mental health disorders n(%)
Setting									
Urban/mostly urban	76	43 (57)	**11 (14)**	40 (53)	19 (25)	**58 (78)**	**41 (58)**	32 (43)	38 (51)
Rural/mostly rural	19	11 (58)	**7 (37)**	12 (63)	9 (47)	**6 (33)**	**4 (24)**	6 (33)	5 (29)
Level of care									
Regional, provincial, or university hospital	53	27 (51)	**5 (9)**	28 (53)	14 (26)	**42 (81)**	**32 (65)**	**26 (50)**	**34 (68)**
District hospital	10	7 (70)	**6 (60)**	8 (80)	6 (60)	**6 (60)**	**5 (50)**	**6 (60)**	**5 (50)**
Health centre	32	20 (63)	**7 (22)**	16 (50)	8 (25)	**16 (53)**	**8 (28)**	**6 (20)**	**4 (13)**
Patients treated at HIV programme									
Adults only	17	**11 (65)**	1 (6)	**10 (59)**	2 (12)	**15 (94)**	**12 (80)**	**12 (75)**	**13 (81)**
Children/adolescents only	17	**5 (29)**	3 (18)	**3 (18)**	4 (24)	**13 (77)**	**10 (59)**	**7 (41)**	**9 (53)**
Adults and children/adolescents	61	**38 (62)**	14 (23)	**39 (64)**	22 (36)	**36 (61)**	**23 (41)**	**19 (32)**	**21 (36)**
Sector									
Public	84	49 (58)	18 (21)	46 (55)	25 (30)	61 (74)	**44 (56)**	**37 (46)**	**43 (53)**
Private	11	5 (45)	0	6 (55)	3 (27)	3 (30)	**1 (11)**	**1 (9)**	**0**
IeDEA region									
Asia‐Pacific	28	12 (43)	3 (11)	11 (39)	8 (29)	21 (75)	19 (68)	16 (57)	18 (64)
Caribbean, Central and South America	8	5 (63)	0	5 (63)	0	7 (88)	4 (57)	4 (57)	4 (50)
Central Africa	10	7 (70)	5 (50)	5 (50)	4 (40)	8 (89)	5 (56)	2 (20)	5 (50)
East Africa	21	10 (48)	4 (19)	12 (57)	9 (43)	8 (42)	6 (32)	8 (41)	7 (37)
Southern Africa	21	14 (67)	5 (24)	16 (76)	5 (24)	14 (67)	7 (37)	5 (24)	5 (26)
West Africa	7	6 (86)	1 (14)	3 (43)	2 (29)	6 (86)	4 (67)	3 (43)	4 (57)

a Differences that were significant at *p* < 0.05 level of significance are highlighted in bold. Percentages are computed using the number of sites with a non‐missing value.

## 4 Discussion

We surveyed 95 HIV treatment sites across 29 LMICs to assess practices for MSD screening and management. Our analyses suggest that gaps in screening for MSDs in HIV sites exist, especially in paediatric clinics and in screening for PTSD in urban settings and middle‐income countries. Gaps in management of MSDs were most commonly reported in rural settings, paediatric clinics, health centres, private sector facilities and in lower middle‐income countries.

These findings highlight the need for additional attention to the mental health needs of children and adolescents living with HIV and integration of mental health services into paediatric HIV treatment programmes. While data on the prevalence of MSDs among children and adolescents living with HIV in LMICs are limited, research indicates that children and adolescents living with HIV experience increased risk of MSDs compared to non‐infected peers 23, 24. Similar to adults, MSDs among children and adolescents are associated with suboptimal HIV treatment outcomes 25, 26, 27. Additional research is needed on how to effectively integrate MSD services into paediatric HIV treatment programmes in LMICs.

Findings also illustrated disparities in access to MSD treatment between urban and rural communities. Implementation research is needed to better understand how to integrate evidence‐based MSDs services into already overburdened health systems in LMICs, including in rural settings where on‐site management of MSDs appears to be less available. Additional in‐depth survey data are needed from rural, peripheral sites to better prioritize MSD integration needs in these settings, particularly as the majority of sites in the current analysis were in urban settings.

A substantial minority of sites reported on‐site management of, but not screening for, depression, PTSD, or other mental health disorders. Screening in the absence of on‐site management of MSDs was also prevalent, particularly for SUDs. Most sites reported that they did not screen for PTSD or other mental health disorders. A substantial minority also reported not screening for depression or SUDs. Among sites that reported MSD screening, most reported screening symptomatic patients. Limited integration of routine MSDs screening and management may be due to limited resources or staff members training or capacity and may be influenced by health system governance and support for comprehensive care delivery models 28, 29. Such practices may also reflect concern that routine screening could overwhelm fragile, underfunded health systems. It is possible that some clinics may not fully appreciate the level of unmet need for MSD care among their patient population. On‐site MSD management in the absence of screening may occur in health facilities that provide separate MSD and HIV care, but in which MSD screening has not been integrated into HIV care or in clinics whose capacity for on‐site MSD management remains limited, reflecting concerns that screening could overwhelm MSD management capacity. A lack of MSDs screening and management in many clinics highlights a current shortcoming of HIV service delivery. Such situations undermine the potential for success in achieving UNAIDS 90‐90‐90 targets 30.

A lack of written guidelines for screening and management of MSDs was also common among sites assessed. Implementation of evidence‐based guidelines for MSD screening and management should be prioritized. Almost half of sites reported not having SSRIs available on site. Psychiatric medications offer a critical treatment option for individuals with MSDs. Strategies to increase access to psychiatric medications in HIV settings, prescribed and managed by trained professionals, should be explored.

Future research should examine potentially modifiable multi‐level barriers and facilitators, including structural, social and economic barriers and facilitators to integrating MSD interventions into HIV care. Greater understanding is needed as to why some HIV care settings report having integrating MSD screening or treatments while others have not.

As access to ART and life expectancy of PLWHA increase, the burden and impact of NCDs among PLWHA, including MSDs, has gained increased attention 12. Using existing HIV care platforms in LMIC to integrate NCD care into HIV and primary care will be critical to meet NCD‐related needs of PLWHA.

The mhGAP provides guidance for how to integrate MSD care into primary care settings 14. Research is needed to better understand how mhGAP recommendations can be most effectively implemented into HIV care settings in LMICs. Future research should examine access to and quality of MSDs services provided at HIV treatment programmes and the extent to which such services are evidence‐based and associated with improved MSD and HIV care outcomes. Research has demonstrated the effectiveness of collaborative care models to integrate MSD treatment into primary care 31, 32. While limited, existing research indicates that integrating MSD care into HIV care in LMICs has the potential to improve mental health and HIV treatment outcomes 33, 34. Additional research on the effectiveness of integrating MSD and HIV care is warranted.

This study has limitations worth noting. IeDEA‐participating HIV treatment sites were selected using a stratified random sampling strategy. While they may be representative of IeDEA, participating sites may not be representative of all sites or HIV treatment programmes in a particular country or region. This analysis relied on reports from health facility staff members regarding screening and management of MSDs at participating clinics. Service access, delivery and quality were not independently verified by direct observation or review of medical records and may be over‐ or under‐represented. This study did not assess specific approaches to MSD screening or management. Wide variability likely exists in the quality and procedures related to MSD screening and management in HIV clinics. Future research should assess how MSD screening is conducted, including the use of validated MSD screening tools, how MSD management is conducted in response to positive MSD screens and the quality of such procedures.

Our study provides initial data on the extent to which screening and management of MSDs has been integrated into HIV treatment programmes in LMICs. Our findings can inform the design of prospective, cohort studies to improve screening and management of MSDs among PLWHA. Future studies should advance understanding of multi‐level barriers to successful integration of MSDs into HIV care in LMICs and identification of optimal implementation strategies to scale and sustain integrated MSD and HIV care.

## Competing interests

The authors declare that they have no competing interests.

## Authors’ contributions

AMP, CM, CWW, SND and DN designed the survey. CM, CWW, SND and DN coordinated data collection. AMP performed the data analysis and interpretation and drafted the manuscript. AP, CM, CWW, SND, DN, FN, CB, EY, KA, AJ and ADH revised the manuscript. All authors read and approved the final manuscript.

## Supporting information


**Additional File A1.** Funding and acknowledgments.
