# Supplementary material for: Screening and management of mental health and substance use disorders in HIV treatment settings in low‐ and middle‐income countries within the global IeDEA consortium
Source: J Int AIDS Soc. 2018 Mar 30;21(3):e25101. doi: 10.1002/jia2.25101 (PMC5878412; doi:10.1002/jia2.25101)
Supplement: Supplementary file 1 — Additional File A1. Funding and acknowledgments. [file JIA2-21-e25101-s001.docx]

**Additional File A1:** Funding and acknowledgments

**Funding:** Research reported in this publication was supported by the U.S. National Institutes of Health’s National Institute of Allergy and Infectious Diseases, the Eunice Kennedy Shriver National Institute of Child Health and Human Development, the National Cancer Institute, the National Institute of Mental Health, and the National Institute on Drug Abuse, as part of the International Epidemiology Databases to Evaluate AIDS, under the following grant numbers: Asia-Pacific (U01AI069907); the Caribbean, Central and South America (U01AI069923); Central Africa (U01AI096299); East Africa (U01AI069911); Southern Africa (U01AI069924); West Africa (U01AI069919). This research was also supported by NIMH center grant (P30-MH43520), NIMH training grant T32 MH019139, NIAID center grant P30 AI110527 (Tennessee Center for AIDS Research), and NCATS grant UL1 TR000445 (Vanderbilt Institute for Clinical and Translational Research). The Kirby Institute is funded by the Australian Government Department of Health and Ageing, and is affiliated with the Faculty of Medicine, UNSW Australia. The content of this publication is solely the responsibility of the authors and does not necessarily represent the official views of any of the governments or institutions mentioned above.

**The IeDEA Asia-Pacific region: The TREAT Asia Observational Database (TAHOD): Site investigators and study team:** PS Ly* and V Khol, National Center for HIV/AIDS, Dermatology & STDs, Phnom Penh, Cambodia; FJ Zhang* ^‡^, HX Zhao and N Han, Beijing Ditan Hospital, Capital Medical University, Beijing, China; MP Lee*, PCK Li, W Lam and YT Chan, Queen Elizabeth Hospital, Hong Kong, China; N Kumarasamy*, S Saghayam and C Ezhilarasi, Chennai Antiviral Research and Treatment Clinical Research Site (CART CRS), YRGCARE Medical Centre, VHS, Chennai, India; S Pujari*, K Joshi , S Gaikwad and A Chitalikar, Institute of Infectious Diseases, Pune, India; TP Merati*, DN Wirawan and F Yuliana, Faculty of Medicine Udayana University & Sanglah Hospital, Bali, Indonesia; E Yunihastuti*, D Imran and A Widhani, Faculty of Medicine Universitas Indonesia - Dr. Cipto Mangunkusumo General Hospital, Jakarta, Indonesia; J Tanuma*, S Oka and T Nishijima, National Center for Global Health and Medicine, Tokyo, Japan; JY Choi*, Na S and JM Kim, Division of Infectious Diseases, Department of Internal Medicine, Yonsei University College of Medicine, Seoul, South Korea; BLH Sim*, YM Gani, and R David, Hospital Sungai Buloh, Sungai Buloh, Malaysia; A Kamarulzaman*, SF Syed Omar, S Ponnampalavanar and I Azwa, University Malaya Medical Centre, Kuala Lumpur, Malaysia; R Ditangco*, E Uy and R Bantique, Research Institute for Tropical Medicine, Manila, Philippines; WW Wong* †, WW Ku and PC Wu, Taipei Veterans General Hospital, Taipei, Taiwan; OT Ng*, PL Lim, LS Lee and Z Ferdous, Tan Tock Seng Hospital, Singapore; A Avihingsanon*, S Gatechompol, P Phanuphak and C Phadungphon, HIV-NAT/Thai Red Cross AIDS Research Centre, Bangkok, Thailand; S Kiertiburanakul*, A Phuphuakrat, L Chumla and N Sanmeema, Faculty of Medicine Ramathibodi Hospital, Mahidol University, Bangkok, Thailand; R Chaiwarith*, T Sirisanthana, W Kotarathititum and J Praparattanapan, Research Institute for Health Sciences, Chiang Mai, Thailand; P Kantipong* and P Kambua, Chiangrai Prachanukroh Hospital, Chiang Rai, Thailand; KV Nguyen*, HV Bui, DTH Nguyen and DT Nguyen, National Hospital for Tropical Diseases, Hanoi, Vietnam; DD Cuong*, NV An and NT Luan, Bach Mai Hospital, Hanoi, Vietnam; AH Sohn*, JL Ross* and B Petersen, TREAT Asia, amfAR - The Foundation for AIDS Research, Bangkok, Thailand; DA Cooper, MG Law*, A Jiamsakul* and R Bijker, The Kirby Institute, UNSW Australia, Sydney, Australia. * TAHOD Steering Committee member; † Steering Committee Chair; ‡ co-Chair. **TREAT Asia Pediatric HIV Nework: Site investigators and cohorts:** PS Ly*, and V Khol, National Centre for HIV/AIDS, Dermatology and STDs, Phnom Penh, Cambodia; J Tucker, New Hope for Cambodian Children, Phnom Penh, Cambodia; N Kumarasamy*, and E Chandrasekaran, YRGCARE Medical Centre, CART CRS, Chennai, India; DK Wati*, D Vedaswari, and IB Ramajaya, Sanglah Hospital, Udayana University, Bali, Indonesia; N Kurniati*, and D Muktiarti, Cipto Mangunkusumo – Faculty of Medicine Universitas Indonesia, Jakarta, Indonesia; SM Fong*, M Lim, and F Daut, Hospital Likas, Kota Kinabalu, Malaysia; NK Nik Yusoff*‡, and P Mohamad, Hospital Raja Perempuan Zainab II, Kelantan, Malaysia; TJ Mohamed* and MR Drawis, Pediatric Institute, Hospital Kuala Lumpur, Kuala Lumpur, Malaysia; R Nallusamy*, and KC Chan, Penang Hospital, Penang, Malaysia; T Sudjaritruk*, V Sirisanthana, and L Aurpibul, Department of Pediatrics, Faculty of Medicine, and Research Institute for Health Sciences, Chiang Mai University, Chiang Mai, Thailand; R Hansudewechakul*, P Ounchanum, S Denjanta, and A Kongphonoi, Chiangrai Prachanukroh Hospital, Chiang Rai, Thailand; P Lumbiganon*†, P Kosalaraksa, P Tharnprisan, and T Udomphanit, Division of Infectious Diseases, Department of Pediatrics, Faculty of Medicine, Khon Kaen University, Khon Kaen, Thailand; G Jourdain, PHPT-IRD UMI 174 (Institut de recherche pour le développement and Chiang Mai University), Chiang Mai, Thailand; T Puthanakit*, S Anugulruengkit, W Jantarabenjakul and R Nadsasarn, Department of Pediatrics, Faculty of Medicine and Research Unit in Pediatric and Infectious Diseases, Chulalongkorn University, Bangkok, Thailand; K Chokephaibulkit*, K Lapphra, W Phongsamart, and S Sricharoenchai, Department of Pediatrics, Faculty of Medicine Siriraj Hospital, Mahidol University, Bangkok, Thailand; KH Truong*, QT Du, and CH Nguyen, Children’s Hospital 1, Ho Chi Minh City, Vietnam; VC Do*, TM Ha, and VT An Children’s Hospital 2, Ho Chi Minh City, Vietnam; LV Nguyen*, DTK Khu, AN Pham, and LT Nguyen, National Hospital of Pediatrics, Hanoi, Vietnam; ON Le, Worldwide Orphans Foundation, Ho Chi Minh City, Vietnam; AH Sohn*, JL Ross, and C Sethaputra, TREAT Asia/amfAR -- The Foundation for AIDS Research, Bangkok, Thailand; DA Cooper, MG Law*, and A Kariminia, The Kirby Institute, UNSW Australia, Sydney, Australia; (*Steering Committee members; † Current Steering Committee Chair; ‡ co-Chair).

**The IeDEA Caribbean, Central, and South America region: (CCASAnet):  Fundación Huésped, Argentina:** Pedro Cahn, Carina Cesar, Valeria Fink, Omar Sued, Emanuel Dell’Isola, Hector Perez, Jose Valiente, Cleyton Yamamoto; **Instituto Nacional de Infectologia-Fiocruz, Brazil:** Beatriz Grinsztejn, Valdilea Veloso, Paula Luz, Raquel de Boni, Sandra Cardoso Wagner, Ruth Friedman, Ronaldo Moreira; **Universidade Federal de Minas Gerais, Brazil:** Jorge Pinto, Flavia Ferreira, Marcelle Maia;

**Universidade Federal de São Paulo, Brazil:** Regina Célia de Menezes Succi, Daisy Maria Machado, Aida de Fátima Barbosa Gouvêa; **Fundación Arriarán, Chile**: Marcelo Wolff, Claudia Cortes, Maria Fernanda Rodriguez, Gladys Allendes; **Les Centres GHESKIO, Haiti:** Jean William Pape, Vanessa Rouzier, Adias Marcelin, Christian Perodin; **Hospital Escuela Universitario, Honduras**: Marco Tulio Luque.  **Instituto Hondureño de Seguridad Social, Honduras:** Denis Padgett. **Instituto Nacional de Ciencias Médicas y Nutrición Salvador Zubirán, Mexico**: Juan Sierra Madero, Brenda Crabtree Ramirez, Paco Belaunzaran, Yanink Caro Vega. **Instituto de Medicina Tropical Alexander von Humboldt, Peru**: Eduardo Gotuzzo, Fernando Mejia, Gabriela Carriquiry. **Vanderbilt University Medical Center, USA:** Catherine C McGowan, Bryan E Shepherd, Timothy Sterling, Karu Jayathilake, Anna K Person, Peter F Rebeiro, Mark Giganti, Jessica Castilho, Stephany N Duda, Fernanda Maruri, Hilary Vansell.

**The IeDEA Central Africa region: Site investigators and cohorts:** Nimbona Pélagie, ANSS, Burundi; Patrick Gateretse, Jeanine Munezero, Valentin Nitereka, Théodore Niyongabo, Christelle Twizere, Centre National de Reference en Matiere de VIH/SIDA, Burundi; Hélène Bukuru, Thierry Nahimana, CHUK, Burundi; Jérémie Biziragusenyuka, Risase Scholastique Manyundo, HPRC, Burundi; Rogers Ajeh, Mark Benwi, Anastase Dzudie, Akindeh Mbuh, CRENC & Douala General Hospital, Cameroon; Kien Atsu, Tabeyang Mbuh, Bamenda Hospital, Cameroon; Djenabou Amadou, Eric Ngassam, Eric Walter Pefura Yone, Jamot Hospital, Cameroon; Alice Ndelle Ewanoge, Norbert Fuhngwa, Chris Moki, Limbe Regional Hospital, Cameroon; Catherine Akele, Faustin Kitetele, Patricia Lelo, Martine Tabala, Kalembelembe Pediatric Hospital, Democratic Republic of Congo; Emile Wemakoy Okitolonda, Landry Wenzi, Kinshasa School of Public Health, Democratic Republic of Congo; Merlin Diafouka, Martin Herbas Ekat, Dominique Mahambou Nsonde, CTA Brazzaville, Republic of Congo; Adolphe Mafou, CTA Pointe-Noire, Republic of Congo; Jean Claude Dusingize, Gallican Kubwimana, Pacifique Mugenzi, Benjamin Muhoza, Athanase Munyaneza, Emmanuel Ndahiro, Diane Nyiransabimana, Jean d'Amour Sinayobye, Vincent Sugira, Rwanda Military Hospital, Rwanda; Fidele Ntarambirwa, Bethsaida Hospital, Rwanda; Yvonne Tuyishimire, Busanza Health Center, Rwanda; Theogene Hakizimana, Gahanga Health Center, Rwanda; Josephine Ayinkamiye, Gikondo Health Center, Rwanda; Sandrine Mukantwali, Kabuga Health Center, Rwanda; Henriette Kayitesi, Olive Uwamahoro, Kicukuro Health Center, Rwanda; Viateur Habumuremyi, Nyarugunga Health Center, Rwanda; Joyce Mukamana, Masaka Health Center, Rwanda; Chantal Benekigeri, Gilbert Mbaraga, WE-ACTx Health Center, Rwanda.  **Coordinating and Data Centers:** Adebola Adedimeji, Kathryn Anastos, Lynn Murchison, Jonathan Ross, Albert Einstein College of Medicine, USA; Diane Addison, Meg Baker, Ellen Brazier, Heidi Jones, Elizabeth Kelvin, Sarah Kulkarni, Denis Nash, Olga Tymejczyk, Radhika Wikramanayake, City University of New York (CUNY), USA; Batya Elul, Columbia University, USA; Xiatao Cai, Don Hoover, Chunshan Li, Qiuhu Shi, Data Solutions, USA; Marcel Yotebieng, Ohio State University, USA; Mark Kuniholm, University at Albany, State University of New York, USA; Andrew Edmonds, University of North Carolina at Chapel Hill, USA; Stephany Duda; Vanderbilt University School of Medicine, USA; April Kimmel, Virginia Commonwealth University School of Medicine, USA.

**The IeDEA East Africa region: Site investigators and cohorts:** Diero L, Ayaya S, Sang E, MOI University, AMPATH Plus, Eldoret, Kenya; Bukusi E, Charles Karue Kibaara, Elisheba Mutegi, KEMRI (Kenya Medical Research Institute), Kisumu, Kenya; John Ssali, Mathew Ssemakadde, Masaka Regional Referral Hospital, Masaka, Uganda; Mwebesa Bosco Bwana, Michael Kanyesigye, Mbarara University of Science and Technology (MUST), Mbarara, Uganda; Barbara Castelnuovo; John Michael Matovu, Infectious Diseases Institute (IDI), Mulago, Uganda; Fred Nalugoda, Francis X. Wasswa, Rakai Health Sciences Program, Kalisizo, Uganda; G.R. Somi, Joseph Nondi, NACP (National AIDS Control Program) Dar es Salaam, Tanzania; Rita Elias Lyamuya, Francis Mayanga, Morogoro Regional Hospital, Morogoro, Tanzania; Kapella Ngonyani, Jerome Lwali, Tumbi Regional Hospital, Pwani, Tanzania; Mark Urassa, Denna Michael, Richard Machemba, National Institute for Medical Research (NIMR), Kisesa HDSS, Mwanza, Tanzania; Kara Wools-Kaloustian, Constantin Yiannoutsos, Rachel Vreeman, Beverly Musick, Indiana University School of Medicine, Indiana University, Indianapolis, IN, USA; Batya Elul, Columbia University, New York City, NY, USA; Jennifer Syvertsen, Ohio State University, Columbus, OH, USA; Rami Kantor, Brown University/Miriam Hospital, Providence, RI, USA; Jeffrey Martin, Megan Wenger, Craig Cohen, Jayne Kulzer, University of California, San Francisco, CA, USA; Paula Braitstein, University of Toronto, Toronto, Canada

**The IeDEA Southern Africa region: Site investigators and cohorts:** Gary Maartens, Aid for AIDS, South Africa; Michael Vinikoor, Centre for Infectious Disease Research in Zambia (CIDRZ), Zambia; Monique van Lettow, Dignitas, Malawi; Robin Wood, Gugulethu ART Programme, South Africa; Nosisa Sipambo, Harriet Shezi Clinic, South Africa; Frank Tanser, Africa Centre for Health & Population Studies (Hlabisa), South Africa; Jonathan Euvrard, Khayelitsha ART Programme, South Africa; Geoffrey Fatti, Kheth’Impilo, South Africa; Sam Phiri, Lighthouse Clinic, Malawi; Cleophas Chimbetete, Newlands Clinic, Zimbabwe; Karl Technau, Rahima Moosa Mother and Child Hospital*,* South Africa; Brian Eley, Red Cross Children's Hospital, South Africa; Josephine Muhairwe, SolidarMed Lesotho; Anna Jores, SolidarMed Mozambique; Kamelia Kamenova, SolidarMed Zimbabwe, Matthew P Fox, Themba Lethu Clinic, South Africa; Hans Prozesky, Tygerberg Academic Hospital, South Africa. **Data centers:** Nina Anderegg, Marie Ballif, Julia Bohlius, Cam Ha Dao Ostinelli, Frédérique Chammartin, Matthias Egger, Lukas Fenner, Per von Groote, Andreas Haas, Taghavi Katayoun, Eliane Rohner, Adrian Spörri, Gilles Wandeler, Elizabeth Zaniewski, Kathrin Zürcher**,** Institute of Social and Preventive Medicine, University of Bern, Switzerland; Andrew Boulle, Morna Cornell, Mary-Ann Davies, Nicola Maxwell, Leigh Johnson, Mmamapudi Kubjane, Patience Nyakato, Gem Patten, Michael Schomaker, Priscilla Tsondai, Victoria Iyun, School of Public Health and Family Medicine, University of Cape Town, South Africa.

**The IeDEA West Africa region: Site investigators and cohorts:** Adult cohorts: Marcel Djimon Zannou, CNHU, Cotonou, Benin; Armel Poda, CHU Souro Sanou, Bobo Dioulasso, Burkina Faso; Fred Stephen Sarfo & Komfo Anokeye Teaching Hospital, Kumasi, Ghana; Eugene Messou, ACONDA CePReF, Abidjan, Cote d’Ivoire; Henri Chenal, CIRBA, Abidjan, Cote d’Ivoire; Kla Albert Minga, CNTS, Abidjan, Cote d’Ivoire; Emmanuel Bissagnene, & Aristophane Tanon, CHU Treichville, Cote d’Ivoire; Moussa Seydi, CHU de Fann, Dakar, Senegal; Akessiwe Akouda Patassi, CHU Sylvanus Olympio, Lomé, Togo.

Pediatric cohorts: Sikiratou Adouni Koumakpai-Adeothy,_CNHU, Cotonou, Benin; Lorna Awo Renner, Korle Bu Hospital, Accra, Ghana; Sylvie Marie N’Gbeche, ACONDA CePReF, Abidjan, Ivory Coast; Clarisse Amani Bosse, ACONDA_MTCT+, Abidjan, Ivory Coast; Kouadio Kouakou, CIRBA, Abidjan, Cote d’Ivoire; Madeleine Amorissani Folquet, CHU de Cocody, Abidjan, Cote d’Ivoire; François Tanoh Eboua, CHU de Yopougon, Abidjan, Cote d’Ivoire; Fatoumata Dicko Traore, Hopital Gabriel Toure, Bamako, Mali; Elom Takassi, CHU Sylvanus Olympio, Lomé,Togo; **Coordinating & data centers:** François Dabis, Elise Arrive, Eric Balestre, Renaud Becquet, Charlotte Bernard, Shino Chassagne Arikawa, Alexandra Doring, Antoine Jaquet, Karen Malateste, Elodie Rabourdin, Thierry Tiendrebeogo, ADERA, Isped & INSERM U1219, Bordeaux, France. Sophie Desmonde, Julie Jesson, Valeriane Leroy, Inserm 1027, Toulouse, France

Didier Koumavi Ekouevi, Jean-Claude Azani, Patrick Coffie, Abdoulaye Cissé, Guy Gnepa, Apollinaire Horo, Christian Kouadio, Boris Tchounga, PACCI, CHU Treichville, Abidjan, Côte d’Ivoire
